# Development and validation of enzyme-linked immunosorbent assays for the serodiagnosis of canine bartonelloses

**DOI:** 10.1128/jcm.00267-25

**Published:** 2025-11-26

**Authors:** Pradeep Neupane, Ricardo G. Maggi, Manoj Basnet, Richard T. Marconi, Edward B. Breitschwerdt

**Affiliations:** 1 Department of Clinical Sciences, College of Veterinary Medicine, North Carolina State University 6798 , Raleigh, North Carolina, USA; 2 FedEx Corporation, Collierville, Tennessee, USA; 3 Department of Microbiology and Immunology, Virginia Commonwealth University Medical Center 72054, Richmond, Virginia, USA; University of California, Davis, Davis, California, USA

**Keywords:** *Bartonella henselae*, proteins, serology, ELISA

## Abstract

*Bartonella* species, emerging vector-borne pathogens of dogs, are increasingly associated with severe, chronic sequelae, as well as potentially life-threatening diseases, such as endocarditis and myocarditis. Diagnosis of bartonelloses is mainly based on PCR, culture, and serological assays. Despite molecular and biotechnological advances, serological assays employing immunofluorescence antibody (IFA), Western blotting, and enzyme-linked immunosorbent assay (ELISA) technologies have encountered diagnostic limitations, primarily due to poor sensitivity. Using sera from *Bartonella*-infected and naïve dogs, we applied an immunoproteomic approach to develop a reliable ELISA assay for the diagnosis of bartonelloses in dogs. Five recombinant *Bartonella henselae* immunodominant proteins (rATP-β, rGroEL, rLemA, rSucB, and rVirB5) were tested in an ELISA format. Sensitivity and specificity of each protein were calculated based on an imperfect reference IFA assay. Dogs comprised Group I: 36 *Bartonella* spp. naturally infected dogs (all *B. henselae* IFA seroreactive) and Group II: 34 *Bartonella* spp. PCR-negative and IFA-negative dogs. Based upon the ELISA seroreactivity results, rATP-β and rGroEL represented the most sensitive and specific candidate peptide targets for utilization in a canine diagnostic ELISA assay. rGroEL resulted in the sensitivity of 83% and specificity of 94% at an optical density (OD) cutoff value of 0.439 and area under curve (AUC) score of 0.93 (95% CI 0.87–0.99), while the sensitivity and specificity of rATP-β were 69% and 94%, respectively, at a cutoff value of 0.565. The combination of rATP-β with rGroEL resulted in an improved sensitivity of 88% and specificity of 92% at an OD cutoff value of 0.505. A receiver operating characteristic curve analysis for the rATP-β plus rGroEL yielded an AUC score of 0.899 (95% CI 0.809–0.989). Combining rATP-β with rGroEL could potentially further improve both the diagnostic sensitivity and specificity of an ELISA assay for the diagnosis of canine bartonelloses.

**IMPORTANCE:**

*Bartonella* species are associated with a wide spectrum of clinical signs and life-threatening diseases in dogs. There is an increased risk of *Bartonella* transmission from dogs to dogs, and from dogs to other animals and humans via vectors, such as ticks, fleas, or direct contact with infected clinical specimens. Due to the poor sensitivity of currently available molecular and serological assays, the diagnosis, treatment, and prevention of *Bartonella* infection in dogs remains challenging. Developing a reliable serodiagnostic assay is essential for the clinical management of canine bartonelloses, a group of infections caused by *Bartonella* species in dogs. Rapid diagnosis and timely treatment of canine bartonelloses could save the lives of thousands of dogs worldwide each year. This study provides key insights into the design of diagnostic tools utilizing *Bartonella henselae* proteins that show promise as serological markers to improve the diagnosis of canine bartonelloses.

## INTRODUCTION

*Bartonella* species are emerging vector-borne pathogens of dogs that are transmitted to mammals through fleas, keds, lice, mites, sand flies, and potentially ticks (1, 2). Examples of known diseases caused by *Bartonella* infections in dogs include lymphadenopathy, chronic bacteremia, endocarditis, bacillary angiomatosis, bacillary peliosis, granulomatous hepatitis, myocarditis, and cutaneous vasculitis (1–4). The increase in the number of recognized infections reported in dogs caused by *Bartonella* spp. over the last decade imposes a previously unrecognized economic and medical burden for animal health services (2, 5, 6). Currently, culture, PCR, and serology are the mainstays for diagnosing bartonelloses in animals (2, 4, 7). Detection of anti-*Bartonella* antibodies in serum by immunofluorescence antibody (IFA) assay, enzyme-linked immunosorbent assay (ELISA), and Western blotting (WB) has been widely used for seroepidemiological and diagnostic purposes, with IFA testing considered the “gold standard” (8–14). However, the diagnostic accuracy of these assays remains poor or incompletely characterized in the context of chronic infection. The management, control, and epidemiological understanding of bartonelloses pose significant challenges due to the absence of cost-effective, sensitive, and rapid diagnostic tests. Previous studies have suggested that sensitivity and potential antigenic cross-reactivity are recurring challenges in ELISA development efforts for serodiagnosis of *Bartonella* infection in dogs and humans (12–15). Therefore, there is a need to develop a rapid and reliable serodiagnostic assay for the detection of serum anti-*Bartonella* antibodies in dogs.

Genetically diverse *Bartonella* spp. and strains have been isolated from numerous hosts, including humans, bats, cats, dogs, rabbits, rodents, horses, cattle, and a large spectrum of insect vectors (1, 2, 5, 7). More than 50 *Bartonella* spp. have been detected in animals or humans, among which at least six *Bartonella* spp. (*B. henselae, B. vinsonii, B. rochalimae, B. clarridgeiae, B. elizabethae,* and *B. quintana*) are known to infect dogs (1–3). Exposure of dogs to genetically diverse *Bartonella* spp. and strains, differences in the duration of infection, and variation in host response, as well as the ability of *Bartonella* spp. to evade the host immune system has likely contributed to variation in the sensitivity and specificity results reported for *Bartonella* ELISA, IFA, and WB modalities. Our laboratory previously reported improved sensitivity of *B. henselae* WB for documenting *Bartonella* exposure in dogs compared to a lower sensitivity for *B. henselae* IFA (53% versus 22%, respectively), when testing serum samples from *Bartonella* PCR-positive naturally infected dogs (*n* = 36) and *Bartonella* PCR-negative and IFA-negative dogs (*n* = 34) (8). Although lacking in sensitivity, *B. henselae* IFA has a high degree of specificity when testing sera from sick dogs (9, 16). To a very limited extent, both ELISA and WB have been used in canine seroepidemiological and serodiagnostic studies to date, with both serodiagnostic modalities also leading to false-negative results.

Several *Bartonella* immunodominant antigens appear to be suitable for the development of an accurate serodiagnostic assay; however, no single antigen has demonstrated both high sensitivity and specificity for the serodiagnosis of *Bartonella* infections in sick dogs or human patients (10, 13, 15, 17, 18). In the present study, we focused on the evaluation of several ELISAs for the detection of anti-*Bartonella* antibodies, using sera from dogs naturally infected with *Bartonella* spp. compared to their cohort controls (sera from *Bartonella* spp. PCR-negative and IFA-negative dogs). We specifically describe the cloning, expression, purification, and preliminary diagnostic utility of five recombinant *B. henselae* antigenic targets: ATP-β, ATP synthase subunit beta, GroEL; a heat shock protein, LemA; a membrane protein, SucB; dihydrolipoamide succinyltransferase protein, and VirB5; a putative component of the type IV secretion system. In this study, we selected two *B. henselae* proteins (SucB and VirB5) previously identified through literature mining (19–22) and three additional *B. henselae* immunodominant proteins (ATP-β, GroEL, and LemA) identified in our previous WB study (8), all of which have potential for diagnosing bartonelloses.

Our study objectives were as follows: (i) to evaluate the sensitivity and specificity of five *B. henselae* immunodominant recombinant proteins (rATP-β, rGroEL, rLemA, rSucB, and rVirB5) by ELISA using IFA as a “gold-standard” reference assay, and (ii) to develop a novel ELISA that can be employed for the diagnosis of infection with diverse *Bartonella* species and strains.

## MATERIALS AND METHODS

### Bacterial strains and plasmids

The strains and plasmids used in this study are listed in Table 1.

**TABLE 1:** Bacterial strains and plasmids used in this study for cloning, expression, or purification of recombinant *B. henselae* proteins

Strains	Plasmids	Description	Source or reference
	Champion pET200 Directional TOPO Expression Kit	Simplified, efficient Directional TOPO cloning expression system	Invitrogen
*Bartonella henselae* San Antonio 2		Feline origin	(8)
One Shot TOP10 chemically competent *Escherichia coli*		F- mcrA Δ(mrr-hsdRMS-mcrBC) Φ80lacZΔM15 Δ lacX74 recA1 araD139 Δ( araleu)7697 galU galK rpsL (StrR) endA1 nupG	Invitrogen
BL21 Star (DE3) one Shot Chemically competent *Escherichia coli*		F-ompT hsdSB (rB-, mB-) galdcmrne131 (DE3)	Invitrogen
BL21 (DE3)		F-ompT hsdS_B_(r_B_^–^ m_B_^–^) gal dcm (DE3)	Novagen

### Dogs’ serum samples for evaluation of rATP-β-, rGroEL-, rLemA-, rSucB, and rVirB5-based ELISA

Seventy archived sera from dogs previously tested at the North Carolina State University, College of Veterinary Medicine, Vector Borne Diseases Diagnostic Laboratory (NCSU-CVM-VBDDL), or the Intracellular Pathogens Research Laboratory, NCSU-CVM (NCSU-CVM-IPRL) were selected for comparative ELISA testing utilizing each of the purified recombinant *B. henselae* immunodominant proteins. *Bartonella* spp. PCR, *Bartonella* spp. IFA, and other vector-borne canine pathogens testing results for these dogs are summarized in Table S1. Serum samples were categorized into two groups to assess sensitivity and specificity as previously described (15). All sera were stored frozen at −80°C after being submitted to the NCSU-CVM-VBDDL for diagnostic testing between 2016 and 2020. The following Institutional Animal Care and Use approved statement appears on the NCSU-CVM-VBDDL test request form: As a veterinary research laboratory, we reserve the right to use archived samples for research purposes, always respecting the privacy rights of the contributing animal, owner, and veterinarian.

In brief, Group I consisted of 36 stored frozen serum samples from *Bartonella* spp. naturally infected dogs as described previously (15). All 36 Group I dogs were *B. henselae* IFA seropositive: 1 dog had a *B. henselae* IFA IgG titer of 1:64, whereas the remaining 35 dogs had a *B. henselae* IFA IgG titer of ≥1:128. A cutoff titer of ≥1:64 was used to define an IFA seropositive titer. Of the 36 Group I dogs, three dogs were PCR-positive for *Bartonella* spp.; two dogs were PCR-positive for *B. vinsonii,* and one dog was PCR-positive for *B. henselae*. Group II consisted of 34 dogs for which diagnostic testing in the NCSU-CVM-VBDDL and NCSU-CVM-IRPL did not provide evidence of exposure to or infection with a *Bartonella* spp. These sera were used to partially assess the specificity of ELISA assays. These sera were IFA negative (titers < 1:16) to the three *Bartonella* spp. (*B. henselae San Antonio* type 2 (SA2)*, B. vinsonii* subsp. *berkhoffii* genotype I*,* and *B. koehlerae*). All 34 Group II dogs were PCR negative after whole blood DNA extraction for *Bartonella* spp.

### Identification of *B. henselae* immunodominant proteins recognized by sera from experimentally and naturally infected dogs

In our previous WB study (8), six *B. henselae* proteins (proteins of 13, 17, 29, 50, 56, and 150 kDa) appeared to represent *Bartonella*-relevant immunodominant antigens as recognized by a majority of experimentally challenged dogs and dogs naturally infected with *Bartonella* spp. These *B. henselae* immunodominant proteins were identified using an in-house matrix-assisted laser desorption ionization time-of-flight mass spectrometry (MALDI-TOF MS, ABSCIEX TOF/TOF 5800 mass spectrometer) as previously described (23) in Dr. Timothy Haystead’s Laboratory in the Department of Pharmacology and Cancer Biology at the School of Medicine, Duke University, Durham, North Carolina. MALDI-MS/MS data were acquired using the AB Sciex 5800 TOF/TOF Mass Spectrometer (AB Sciex, Framingham, MA). Peptide mass fingerprint and peptide sequence data were resolved by the SPROT (UNIPROT) and NCBI databases using the Mascot search engine.

**Fig 1:**
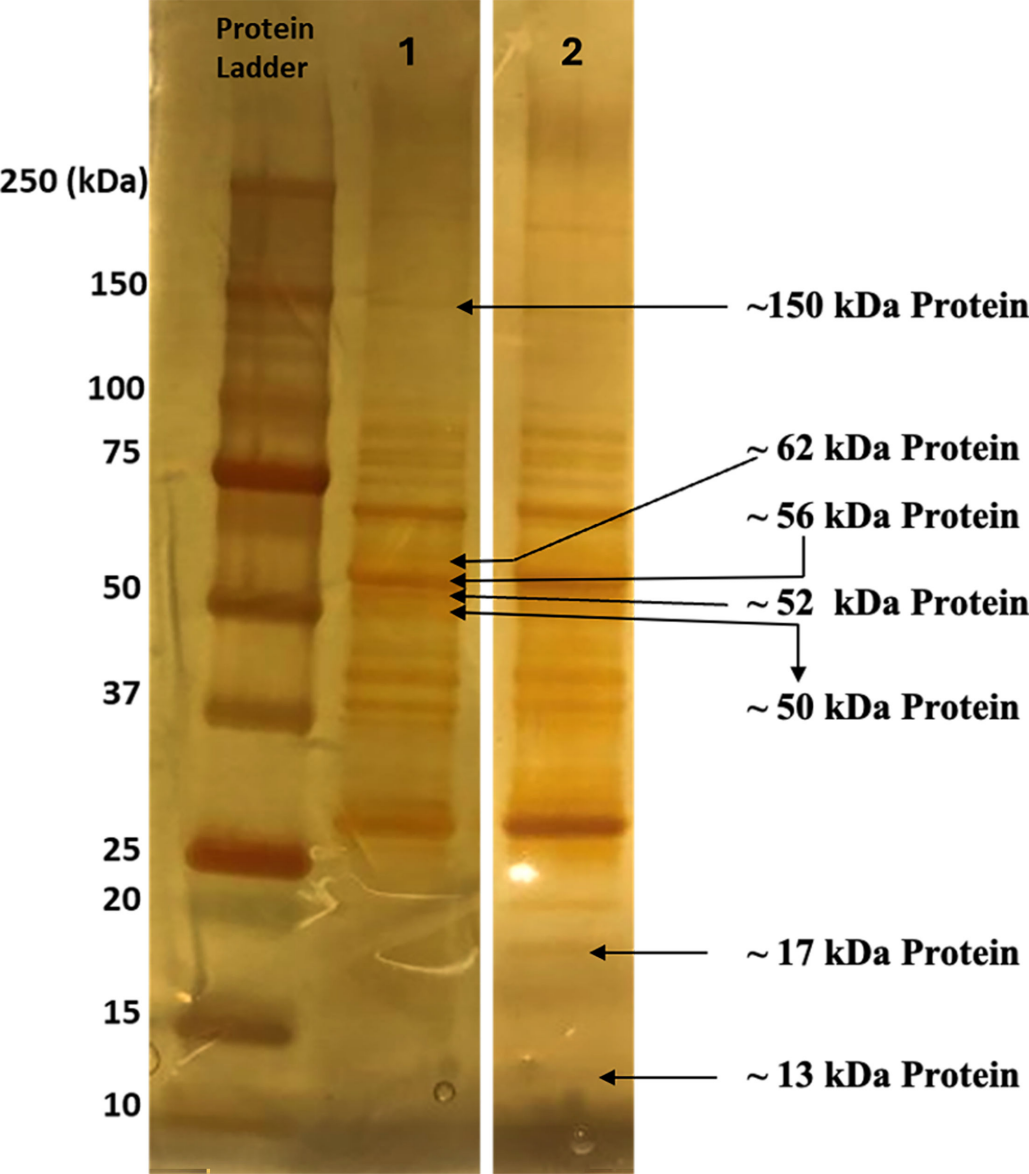
Identification of *B. henselae* immunodominant proteins by the MALDI-TOF MS. The majority of experimentally and naturally infected dogs recognized *Bartonella* species proteins of molecular weights 13, 17, 50, 56, and 150 kDa; thus, these proteins appeared to be *Bartonella*-relevant immunodominant proteins (Neupane, P., Sevala, S., Balakrishnan, N., Marr, H., Wilson, J., Maggi, R., Birkenheuer, A., Lappin, M., Chomel, B., and Breitschwerdt, E.B., 2020). Validation of *B. henselae* western immunoblotting for serodiagnosis of bartonelloses in dogs. *Journal of Clinical Microbiology*, 58(4), pp.e01335-19. For MALDI-TOF MS analysis, heat-denatured *B. henselae* SA2 whole-cell proteins were separated by SDS-PAGE followed by silver staining of the gel (lanes 1 and 2) and MS analysis of the excised immunodominant protein bands.

In brief, heat-denatured *B. henselae* SA2 whole-cell proteins were separated by sodium dodecyl sulfate-polyacrylamide gel electrophoresis (SDS-PAGE) in Criterion precast gels, using 4%–15% gradient polyacrylamide Tris-glycine precast midigels (Bio-Rad, Hercules, CA) as previously described (8). Gel was fixed and silver stained according to published protocols. Each band representing immunodominant proteins of 13, 17, 50, 56, and 150 KDa (Fig. 1) was manually excised from the gel and in-gel digested with trypsin (0.6 µg). Tryptic peptides were subjected to MALDI-MS on an ABSCIEX TOF/TOF 5800 mass spectrometer. In addition, bands representing proteins of molecular weight 52 and 62 KDa were excised and subjected to the same spectrometer as described above for LC-MS/MS. After intradermal inoculation of *B. henselae,* the 62 kDa was recognized by 30% of experimentally inoculated dogs (8). The 52 kDa protein was closest to the 50 kDa immunodominant protein that was recognized by dog sera in our WB study (8).

Based on peptide sequence and peptide fingerprint data, the *B. henselae* immunodominant proteins identified from each band are summarized in Table 2. All proteins listed achieved the minimum criteria for a single peptide with 99% confidence. The 56 kDa and 150 kDa bands were identified as *B. henselae* GroEL protein (NCBI Reference Sequence WP_011181151.1), a heat shock protein, and DNA-directed RNA polymerase subunit beta (WP_11180534.1), respectively. With the exception of 56 kDa and 150 kDa bands, more than two proteins were identified from each excised band (Table 2). MS analysis of the 13 kDa band failed, probably due to a minimum protein content.

**TABLE 2:** Identification of *B. henselae* immunodominant proteins by the MALDI-TOF MS^*a*^

WB band (kDa)	Protein identification by MALDI-TOF MS
13	N/A
17	LemA Family protein (WP_011180688.1), Invasion-associated protein IalB (WP_034454864), Peptidylpropyl isomerase (WP_011180874.1), 50S ribosomal protein L6 (WP_082250521), and Transcription factor GreA (WP_034447961),
50	ATP Synthase subunit alpha (WP_082251272.1), ATP Synthase subunit beta (WP_011181298.1), and GroEL (WP_011181151.1)
52	GroEL (WP_011181151.1), ATP Synthase subunit beta (WP_011181298.1), and dihydrolipoyl dehydrogenase (WP_082252214.1)
56	GroEL (WP_011181151.1)
62	Succinate dehydrogenase flavoprotein subunit (SdhA; WP_034454472.1) and Peptidylprolyl isomerase (WP_082252249.1)
150	DNA-directed RNA polymerase subunit beta (WP_11180534.1)

^*a*^ All proteins listed achieved the minimum criteria of one peptide with 99% confidence. With the exception of 56 and 150 kDa proteins, more than two proteins were identified from each gel band. MALDI-MS/MS data were acquired using the AB Sciex 5800 TOF/TOF Mass Spectrometer (AB Sciex, Framingham, MA). N/A, not applicable.

### Selection of *B. henselae* immunodominant proteins for ELISA development for the diagnosis of canine bartonelloses

Among the *B. henselae* immunodominant proteins, each protein of a molecular weight of 17, 50, and 56 kDa yielded a sensitivity of ≥35% and specificity of ≥92% for canine bartonelloses as determined by reactivity of these protein bands with sera from naturally infected (*Bartonella* PCR positive) compared to negative control dogs in our previous WB study (8). Of the several proteins identified from 17 kDa and 50 kDa bands, we selected *B. henselae* LemA and rATP-β for this study, based on reactivity patterns of these immunodominant patterns in previous studies (21, 24, 25). *B. henselae* GroEL was the only identified protein from the 56 kDa gel band that was evaluated in this study. Previous studies reported GroEL as a *B. henselae* immunodominant protein that could be a suitable diagnostic marker for bartonelloses (21, 26). Two additional *B. henselae* immunodominant proteins (SucB and VirB5) were selected through literature mining (20–22). These prior studies also suggested that SucB and VirB5 may be potential diagnostic candidates for the diagnosis of bartonelloses. Thus, based upon WB results and literature mining, we elected to evaluate the diagnostic utility of five *B. henselae* recombinant proteins (rATP-β, rGroEL, rLemA, rSucB, and rVirB5) in this study.

### Selection of immunodominant regions of *B. henselae* proteins for ELISAs development for the diagnosis of canine bartonelloses

**Fig 2:**
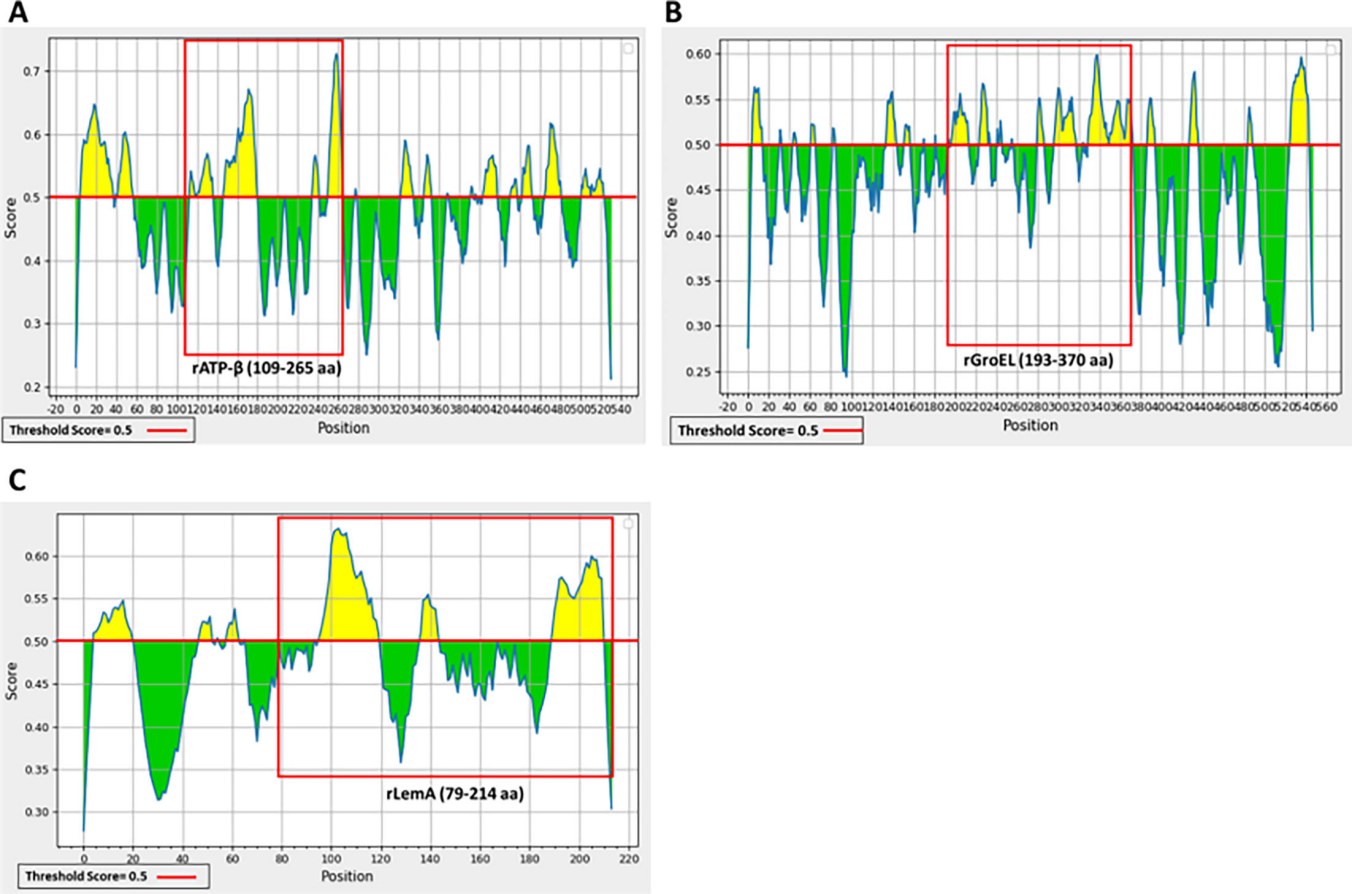
Predicted B-cell epitopes of *B. henselae* F0F1 ATP synthase subunit beta (NCBI Reference Sequence: WP_011181298.1), *B. henselae* GroEL (NCBI Reference Sequence: WP_011181151.1), and *B. henselae* LemA protein (NCBI Reference Sequence: WP_011180688.1) using the IEDB antibody epitope prediction tool (BepiPred 2.0). (**A**) Analysis of *B. henselae* F0F1 ATP synthase subunit beta, (**B**) *B. henselae* GroEL, and (**C**) *B. henselae* LemA. *B. henselae* F0F1 ATP synthase subunit beta (NCBI Reference Sequence: WP_011181298.1; ranging from 109 to 265 amino acids), *B. henselae* GroEL peptide (WP_011181151.1; ranging from 193 amino acid to 370 amino acids), and *B. henselae* LemA protein (NCBI Reference Sequence: WP_011180688.1; ranging from 79 to 214 amino acids) comprised predicted B-cell epitopes with higher scores, which are represented by red boxes. Based on immunoproteomic analysis, these peptide regions of *B. henselae* ATP-β, GroEL, and LemA were selected for cloning, expression, and purification.

To assess the potential diagnostic utility of ATP-β, GroEL, and LemA proteins, identified from our WB study, BepiPred 2.0 with default parameter settings provided by IEDB (Immune-Epitope-Database and Analysis-Resource) was applied to the *B. henselae* ATP-β, GroEL, and LemA protein sequences (27, 28). Based upon B-cell epitope mapping, beta-turn, surface accessibility, antigenicity, and hydrophilicity prediction (http://tools.iedb.org/bcell/), *B. henselae* F0F1 ATP synthase subunit beta (NCBI Reference Sequence WP_082251662.1; ranging from 109 to 265 amino acids), *B. henselae* GroEL (NCBI Reference Sequence: WP_034454894.1; ranging from 193 to 370 amino acids), and *B. henselae* LemA protein (NCBI Reference Sequence: WP_011180688.1; ranging from 79 to 214 amino acids), all comprising high scores for predicted B-cell epitopes, were selected (Fig. 2A through C and Table 3). These immunodominant peptide regions of ATP-β, GroEL, and LemA are represented by the red boxes in Fig. 2, whereas the full-length recombinant SucB and VirB5 proteins were selected for evaluation. The selected regions of ATP-β, GroEL, and LemA, and full-length recombinant SucB and VirB5 used in this study are designated rATP-β, rGroEL, rLemA, rSucB, and rVirB5.

**TABLE 3:** Regions of *B. henselae* F0F1 ATP synthase subunit beta (ATP-β), a heat shock protein (GroEL), a membrane protein (LemA), dihydrolipoamide succinyltransferase protein (SucB), and a putative component of the type IV secretion system (VirB5) that were selected for cloning, purification, and expression in this study^*a*^

Proteins	NCBI Reference Sequence (length; mol mass)	Selected region for cloning (location)	Designated recombinant protein name	Mol. mass (kDa) of selected region	Reference
ATP-β	WP_011181298.1 (531 aa; 56.59 kDa)	157 (109–265 aa)	rATP-β	16.52	This study
GroEL	WP_011181151.1 (547 aa; 57.65 kDa)	178 (193–370)	rGroEL	19.59	This study
LemA	WP_011180688.1 (214 aa; 24.50 kDa)	136 (79–214)	rLemA	14.1	This study
SucB	WP_011181413.1 (406 aa; 43.46 kDa)	406 (1–406)	rSucB	43.65	This study
VirB5	WP_011181130.1 (148 aa; 16.9 kDa)	147 (1–147)	rVirB5	16.9	This study

^*a*^ aa, amino acids.

### Amplification of *B. henselae atpD***,** *groEL***,** *lemA***,** *sucB***,** and *virB5*

The full-length *sucB* and *virB5* and selected regions of *atpD*, *groEL,* and *lemA* genes were PCR-amplified, cloned, and expressed using the *Escherichia coli* expression system (Table 1). Genomic DNA of *B. henselae* SA2 was extracted from a blood agar plate grown with *B. henselae* SA2 using DNeasy Blood and Tissue Kit (Qiagen, Valencia, CA) following the manufacturer’s protocol. The primer sets used for conventional PCR amplification of the *B. henselae* SA2 *atpD***,** *groEL***,** *lemA***,** *sucB***,** and *virB5* genes are listed in Table 4. Conventional PCR was performed in a 25 µL final volume reaction containing 12.5 µL of Q5 High-Fidelity 2X Master mix (New England Biolabs, USA, cat. No. M0492S), 0.2 µL of 100 µM of each forward and reverse primer (IDT-DNA Technology), 7.3 µL of molecular-grade water, and 5 µL of DNA from each sample tested. Genomic DNA from *B. henselae* Houston-1 was used as a positive control. Five microliters of Ultra-Pure, molecular grade water (Genesee Scientific, San Diego, CA, USA) and 5 µL of DNA extracted from *Escherichia coli* were used as negative controls. Conventional PCR was performed in an Eppendorf Mastercycler EP gradient under the following conditions: a single hot-start cycle at 95°C for 2 min followed by 30 cycles of denaturing at 95°C for 30 s, annealing at 58°C for 30 s, and extension at 72°C for 30 s. Amplification was completed by an additional cycle at 72°C for 2 min. Prior to the ligation reaction for cloning, PCR products were purified by gel extraction using PureLink quick gel extraction and PCR purification combo kit (Invitrogen, Carlsbad, CA).

**TABLE 4:** List of the PCR primers used for cloning, expression, and purification of the *B. henselae* San Antonio 2 *atpD, groEL*, *lemA*, *sucB*, and *virB5* genes using Champion pET200 Directional TOPO Expression kit^*a*^

Recombinant proteins	Selected region of proteins for cloning and purification (NCBI Reference Sequence; selected amino acids [aa])	Primers used for PCR amplification and cloning (Sequence 5′ → 3′)
rATP-β	WP_011181298.1; 109–265 aa	BhATP-β−325F **CACC**GCCATGGATACTACCGATGGTCTTG
		BhATP-β−795R TTATCCTTCTGTTGAACCATTGTTGTC
rGroEL	WP_011181151.1; 193–370 aa	BhGroEL-577F **CACC**ATGCAGTTTGATCGTGGATATCTTTC
		BhGroEL-1110R TTAAGCAAGTCTTTCTTGCAATTTTTC
rLemA	WP_011180688.1; 79–214 aa	BhLemA-235F **CACC**GCTACCCATGAACAAGCTGTTTTTAC
		BhLemA-645R TTAATTAAAATTAACCTTCGGTGTTTG
rSucB	WP_011181413.1; 1–406 aa	BhSucB-1F **CACC**ATGACTACTGAAATCCGTGTTCC
		BhSucB-1221R TTACAAGTCAAGAACCAGGCG
rVirB5	WP_011181130.1; 1–147 aa	BhVirB5-1F **CACC**ATGAAAAAATATAGCTTAGTCAC
		BhVirB5-447R CTAAAGTCGGACATCAGATTTTCC

^*a*^ The four nucleotides in bold represent nucleotides that were added at the 5′ end of forward primer to enable directional cloning in the pET200/D-TOPO vector.

### Cloning, expression, and purification of recombinant ATP-β, GroEL, LemA, SucB, and VirB5

The PCR-amplified *atpD*, *groEL, lemA, sucB,* and *virB5* genes were inserted into the Champion pET200 Directional TOPO Expression kit (Invitrogen, Carlsbad, CA) and transformed into *E. coli* chemically competent TOP10 cells. The recombinant plasmids were then purified, and the insert sequences were confirmed by Sanger sequencing (Genewiz, Research Triangle Park, NC). Recombinant plasmid constructs were then transformed into BL21 Star (DE3) (Invitrogen, Carlsbad, CA) and induced at 1 mM IPTG for protein purification. Expression of recombinant products was verified by resolution of total crude protein by SDS-PAGE followed by WB analysis using Pierce 6X-His Epitope-Tag mouse monoclonal antibody (Thermo Scientific, Rockford, IL) and anti-mouse IgG secondary antibody (Rockland, Gilbertsville, PA). IPTG-induced cell pellets were subjected to BugBuster Master Mix (EMD Millipore Corp., Billerica, MA) treatment, and soluble fractions containing His-tagged proteins were purified using HisPur Cobalt spin columns (Thermo Scientific, Rockford, IL) according to the manufacturer’s instructions. Induced recombinant proteins were purified by column chromatography using HisPur Cobalt spin columns according to the manufacturer’s instructions (Thermo Scientific, Rockford, IL). Fractionated proteins were visualized by staining the gel overnight with Bio-safe Coomassie brilliant blue (Bio-Rad, Hercules, CA). Purified recombinant proteins were verified by WB using Pierce 6X-His Epitope-Tag mouse monoclonal antibody (Thermo Scientific, Rockford, IL) and anti-mouse IgG secondary antibody (Rockland, Gilbertsville, PA). Proteins were dialyzed into phosphate-buffered saline (PBS; overnight) using Slide-A-Lyzer Dialysis Cassettes (2-10 kDa MWCO cutoff; Thermo Scientific, Rockford, IL).

### Evaluation of the sensitivity and specificity of five *B. henselae* recombinant immunodominant proteins by ELISA

To evaluate the sensitivity and specificity of purified recombinant ATP-β, GroEL, LemA, SucB, and VirB5, ELISA was performed using sera from Group I (*n* = 36) and II (control group; *n* = 34) dogs. All samples were tested in duplicate without complete blinding. For each ELISA testing run, a set of serum samples was randomly retrieved from dogs in Group I and Group II. All samples were previously labeled with unique identification numbers, and ELISA optical density (OD) readings were recorded without knowledge of their group assignment (Group I vs Group II), that is, without awareness of samples’ *Bartonella* exposure status. ELISA testing and subsequent data analyses were performed carefully to minimize potential bias. Sample identification was only unblinded after the completion of ELISA OD data collection.

Each protein was immobilized in duplicate in ELISA plate wells (500 ng/well) using carbonate buffer as described previously (15). Briefly, ELISA plates were coated with 100 µL of 10 µg/mL recombinant purified proteins in carbonate buffer, pH 9.6, overnight at 4°C. After each incubation time, plates were washed four times using 1× PBS-T washing buffer (1× PBS with 0.05% Tween 20). After washing, plates were blocked with 1.5% nonfat milk powder in 1× PBS (blocking solution) for 2 h at room temperature (RT). After washing, sera from dogs in 1:100 in blocking solution were added and incubated for 1 hour at RT. After washing four times with 1× PBST, plates were incubated with 100 µL of HRP-conjugated goat anti-dog IgG (1:2,000 dilution; Invitrogen, Carlsbad, CA) in blocking solution. The plates were developed with the addition of 50 µL of 1-Step Ultra TMB ELISA substrate solution (Invitrogen, Carlsbad, CA) for 15 min followed by the addition of 2M H_2_SO_4_ to stop the reaction. Absorbance values at 450 nm were measured using a Tecan plate reader.

Sera from dogs naturally infected with *B. henselae* (*B. henselae* IFA titer ≥1: 512) and *Bartonella* PCR-negative and IFA-negative dog sera were used as positive and negative controls, respectively. Plate wells coated with only coating buffer (without protein) were used as blanks to determine the subtract background noise. Based on results from the negative controls, a baseline was established for scoring individual samples as positive or negative for *Bartonella* exposure. The average absorbance value was calculated for each set of duplicate samples. The coefficient of variation between duplicate samples was calculated to evaluate the precision and reproducibility of the assay, particularly intra- and inter-assay variability. ELISA was repeated for samples if the coefficient of variation between the duplicates was greater than 20%. To calculate the sensitivity and specificity of each *B. henselae* immunodominant protein in an ELISA format, the IFA assay was used as the reference (“gold-standard”) serological assay.

### Statistical analysis

Receiver operating characteristic (ROC) analysis was performed with 95% CIs to determine sensitivity, specificity, ELISA cutoff values, and to distinguish reactivity between positive and control groups, as previously described (29, 30). Differences in IgG reactivity to target proteins between infected and control groups were analyzed using the Mann-Whitney *U* test. Optimal density (OD) cutoff values were determined to maximize the Youden index as previously described. Scatterplots of ELISA OD values were generated to determine differences in ELISA seroreactivity between *Bartonella*-infected and control groups. To compare the serological results obtained from ELISA for all groups, the overall percent agreement between *Bartonella* spp. IFA and ELISA was calculated as described previously (31). Scatterplots and ROC curves were generated in the Windows 10 operating system with the help of Anaconda Navigator version 1.9.12. The scatter plots were generated using Python 3. 6.13 in JupyterLab 3.2.1, and the ROC curves were analyzed using R version 3.6.1 in RStudio 1.1.456 (accessed on October 15, 2024). *P* values of less than 0.05 were considered statistically significant.

## RESULTS

### Purification of recombinant *B. henselae* immunodominant (rATP-β, rGroEL, rLemA, rSucB, and rVirB5)

**Fig 3:**
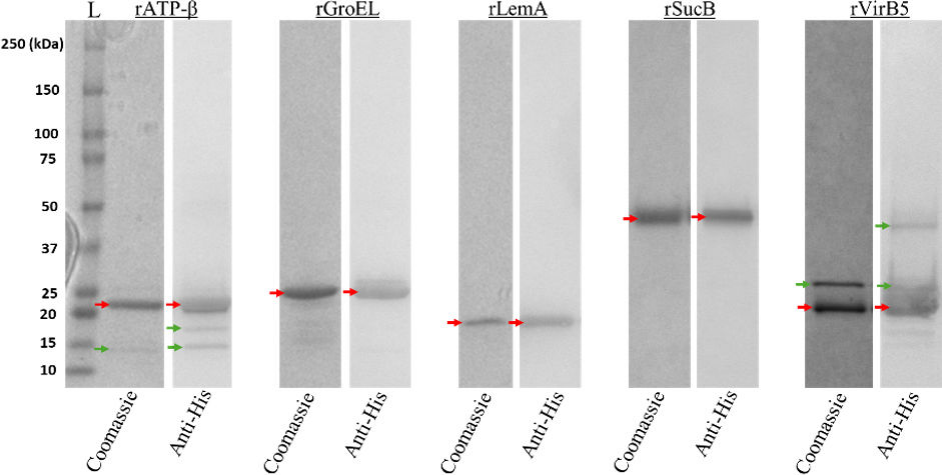
Coomassie-stained SDS-PAGE and WB analysis of purified recombinant proteins (rATP-β, rGroEL, rLemA, rSucB, and rVirB5). WB analysis of purified proteins was performed using mouse anti-His antibody (Thermo Scientific, Rockford, IL) and alkaline-phosphatase-conjugated Goat anti-mouse IgG (Thermo Scientific, Rockford, IL). Predicted molecular mass of recombinant proteins was determined by the coding sequence of the specified gene insert plus fused in-frame with pET200D/TOPO expression system fusion tag (~3 kDa) as indicated by red arrows. Green arrows indicate recombinant proteins in multimeric forms. Coomassie, Coomassie-stained SDS-PAGE; Anti-His, WB analysis; and L, Bio Rad Precision Plus Protein Kaleidoscope Prestained Protein Standard.

Sanger sequencing confirmed in-frame insertion of coding sequences of *atp*-β, *groEL, lemA, sucB,* and *virB5* in the pET200D/TOPO expression system, as determined by amplicon sequence of the plasmid isolated from the respective recombinant *E. coli* BL21 [DE3] clones. Purified proteins were confirmed by Coomassie-stained SDS-PAGE and WB analysis (Fig. 3).

### ELISA reactivity of rATP-β, rGroEL, rLemA, rSucB, and rVirB5

Sensitivity and specificity of each of the five *Bartonella* recombinant proteins were assessed by ELISA testing using sera from Group I, 36 naturally infected, and Group II, 34 control dogs. ELISA OD cutoff values were determined to maximize sensitivity and specificity for each recombinant protein at the maximal Youden index.

**Fig 4:**
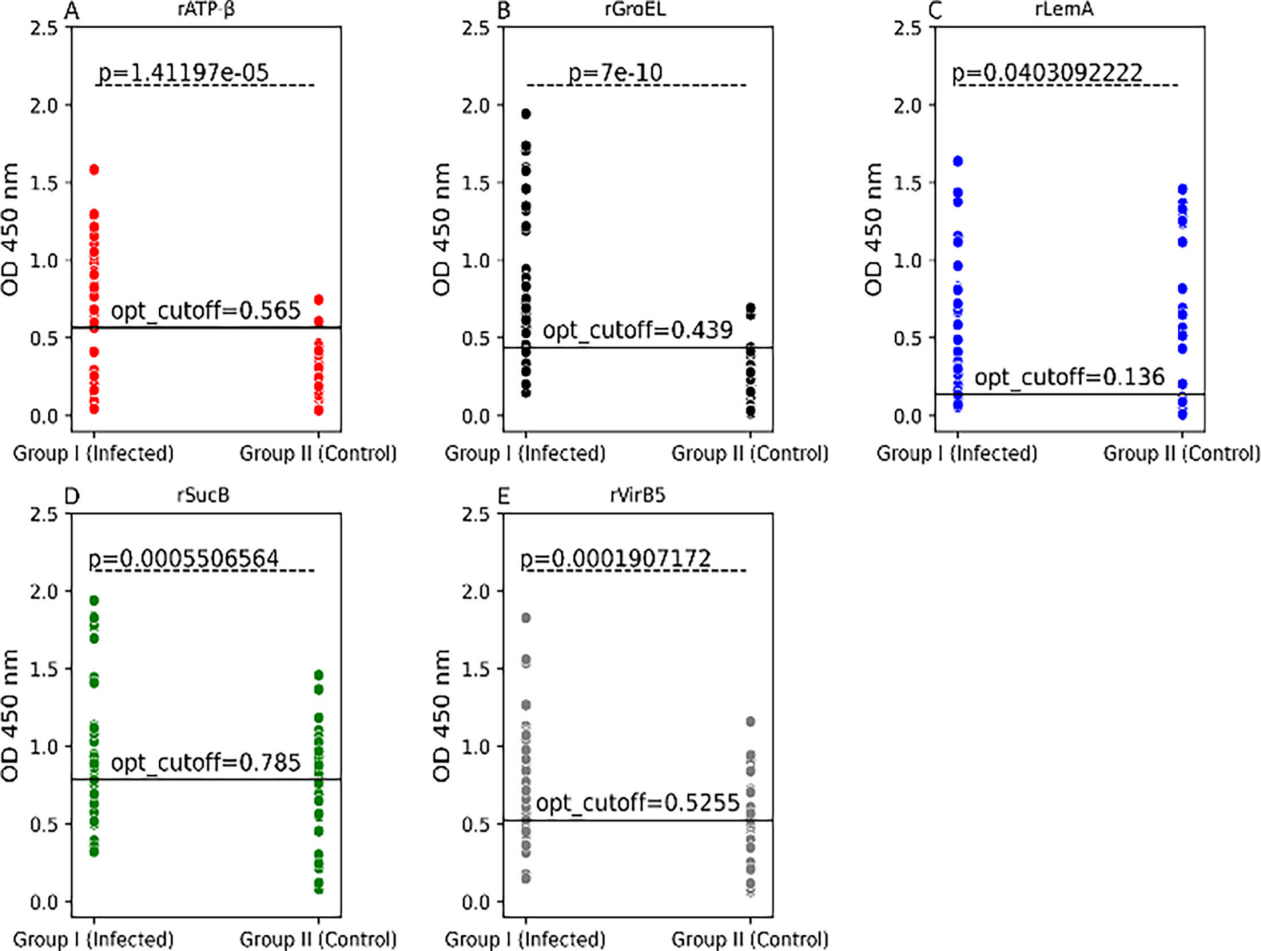
Scatter plots of the ELISA seroreactivity among *Bartonella*-infected and control dogs. ELISA seroreactivity for (**A**) recombinant rATP-β, (**B**) rGroEL, (**C**) rLemA, (**D**) rSucB, and (**E**) rVirB5). For ELISA analysis, sera from naturally infected dogs with *Bartonella* spp. (Group I; *n* = 36) and control dogs (Group II, *n* = 34) were used. Respective *P* values (dotted line) between sample groups are given. OD cutoff values as determined at maximum Youden Index are represented by the black solid line. opt_cutoff, optimal ELISA cutoff value at maximum Youden index.

**Fig 5:**
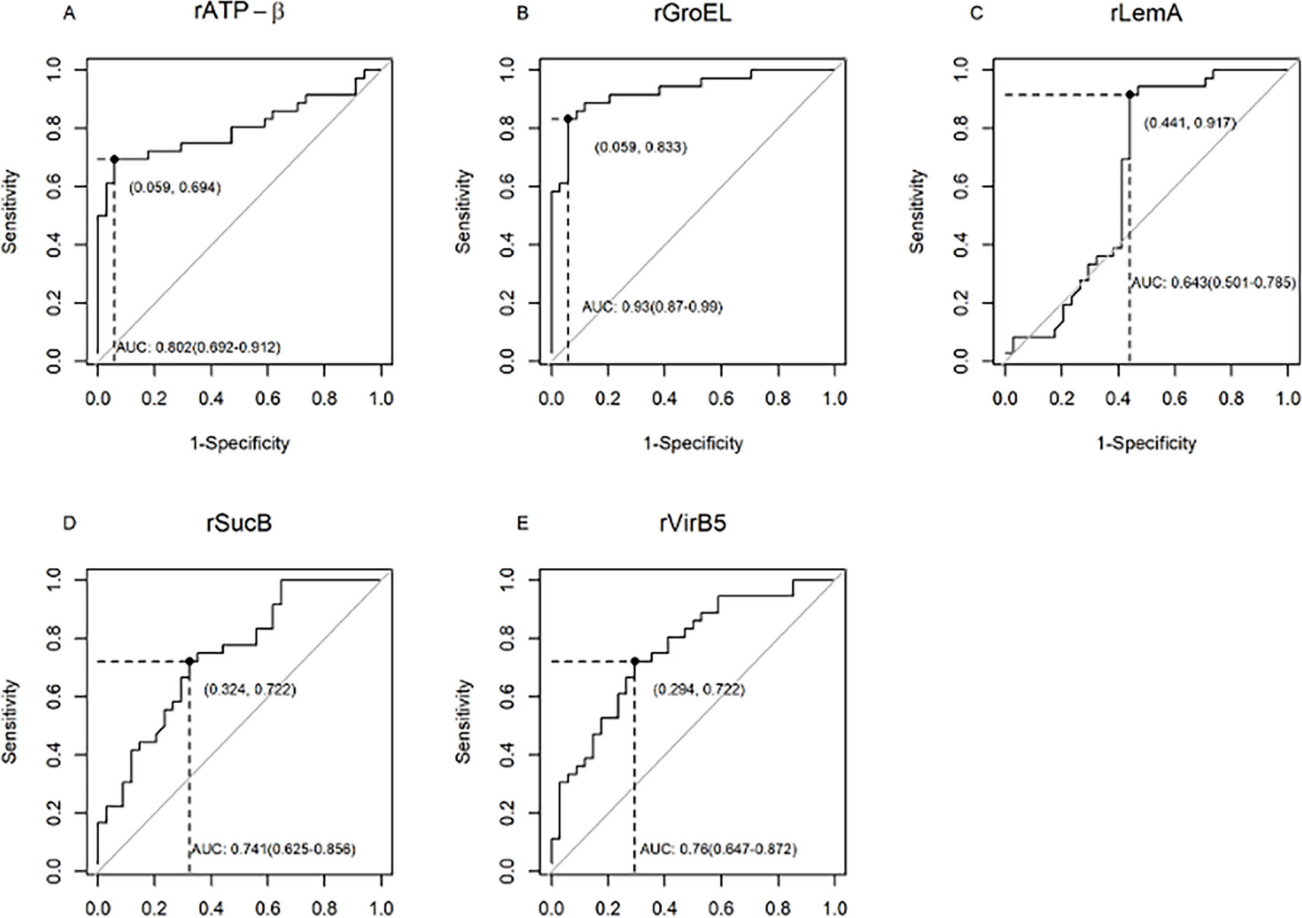
ROC curves with 95% CIs for ELISA seroreactivity of *Bartonella* recombinant proteins for dogs. ROC curves for (**A**) recombinant rATP-β, (**B**) rGroEL, (**C**) rLemA, (**D**) rSucB, and (**E**) rVirB5. Cutoff values were determined to maximize the Youden index. For each recombinant protein, false positives and true positives are shown in parentheses, respectively, at the intersection of the dotted line. AUC, area under curve.

Of the seven *Bartonella* recombinant proteins, rGroEL resulted in an 83% (95% CI 67.19%–93.63%) sensitivity and 94% (95% CI 80.32%– 99.28%) specificity at a cutoff OD value of 0.439 (Fig. 4 and 5). In addition, rGroEL yielded the highest AUC score of 0.93 (95% CI 0.87–0.99) (Fig. 5). The sensitivity and specificity of recombinant rATP-β were 69% (95% CI 51.89%–83.65%) and 94% (95% CI 80.32%–99.28%) at a cutoff value of 0.565. Only two Group II control dogs (0.058%) were reactive to rATP-β at the cutoff value of 0.565. Two Group II dogs were positive for rGroEL at the cutoff of 0.439, one of which was also positive for rATP-β. Of the three Group II control dogs that were reactive to rATP-β or rGroEL at the given cutoff values for respective recombinant proteins, one dog was ELISA positive for both rATP-β and rGroEL proteins. These three dogs were IFA seroreactive to *Rickettsia rickettsii*. The remaining seven of 10 *R. rickettsii* IFA seroreactive Group II dogs tested negative by rATP-β and rGroEL ELISA.

The true-positive and false-positive rates for rVirB5 were 72% and 29%, respectively, and an AUC score for rVirB5 was 0.76 (95% CI, 0.647–0.872). Although the sensitivity of both rLemA and rSucB was >70%, more than 30% of Group II control dogs were reactive to rLemA and rSucB above their respective cutoff values (Fig. 4). Overall, significant binding of IgG was detected in sera from Group I infected dogs as compared with control dogs for all the recombinant proteins (Fig. 4, Mann-Whitney U test, *P* < 0.05). rATP-β- and rGroEL-based ELISA had AUC scores greater than 0.8, whereas AUC scores for all other recombinant proteins were less than 0.75. Based on all the diagnostic parameters tested (sensitivity, specificity, and AUC score), rATP-β and rGroEL represented the optimal candidates for the serodiagnosis of *Bartonella* infection in dogs.

**Fig 6:**
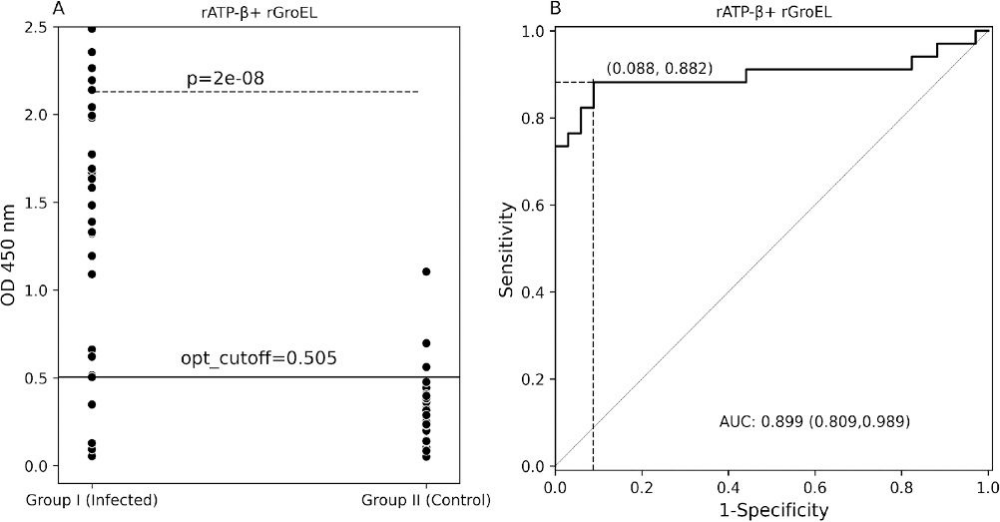
Scatter plots and ROC curve analysis of rATP-β plus rGroEL ELISA seroreactivity among dogs. Scatter plots (**A**) and ROC curve analysis (**B**). Cutoff values were determined to maximize the Youden index. For each recombinant protein, false positives and true positives are shown in parentheses, respectively, at the intersection of dotted lines. opt_cutoff, optimal ELISA cutoff value at maximum Youden index; AUC, area under curve.

Since rATP-β and rGroEL were the most reactive proteins, we used a combination of rATP-β and rGroEL to test 34 Group I (inadequate volume for 2/36 Group I sera) and 34 Group II control dogs (Fig. 6). By combining these two proteins, sensitivity was 88% (95% CI 72.55%–96.70%) and specificity was 92% (95% CI 76.32%–98.14%) at an OD cutoff value of 0.505 at maximum Youden index. At an OD cutoff of 1.195 (trade-off between sensitivity and specificity), sensitivity was 74% (95% CI 55.64%–87.12%) and specificity was 100% (89.72%–100.00%). Of the three rATP-β plus rGroEL ELISA-positive Group II dogs, one was reactive to both rATP-β and rGroEL, one was only reactive to rGroEL, and the remaining dog was not reactive to either rATP-β or rGroEL. A ROC curve analysis for the rATP-β plus rGroEL yielded an AUC score of 0.899 (95% CI 0.809–0.989). Sensitivity and specificity of ELISA using each of the five *Bartonella* recombinant proteins and rATP-β plus rGroEL vary at different cutoff values (trade-off between sensitivity and specificity; Table S2). There was a significant difference (*P* < 0.05) in the rATP-β plus rGroEL reactivity between Group I infected and Group II control dogs (Fig. 6A).

### Comparison of *Bartonella* spp. IFA results and ELISA reactivity for dogs

Since IFA is the most frequently used “gold standard” method for screening anti-*Bartonella* antibodies in serum samples from dogs, we compared diagnostic agreements between *Bartonella spp*. IFA and ELISA results for each immunodominant protein. Comparison between IFA and ELISA results for *Bartonella* infection is summarized in Table 5. Based on the comparison of IFA and ELISA results in dogs, there was substantial agreement (overall 90% agreement; kappa value = 0.794) between *Bartonella* IFA and rATP-β plus rGroEL ELISA. Thirty (83%) of *B. henselae* IFA-positive Group I naturally infected dogs were positive by the rATP-β plus rGroEL ELISA, while 32 of 34 IFA-negative Group II dogs were also negative by the rATP-β plus rGroEL ELISA. The overall proportion agreement between *Bartonella* IFA and rATP-β or rGroEL was substantial (kappa value = 0.63-0.8). Based on the calculation of overall agreement between IFA and ELISA for dogs, the overall proportion agreement was 88% for *Bartonella* IFA and rATP-β ELISA and 81% for *Bartonella* IFA and rGroEL ELISA.

**TABLE 5:** Comparison of *Bartonella* IFA and ELISA results for dogs^*a*^

Dog groups (I and II)		
IFA result	ELISA result	
	rATP-β	
	Positive	Negative
Positive (*n* = 36)	25	11
Negative (*n* = 34)	2	32
	rGroEL	
	Positive	Negative
Positive (*n* = 36)	30	6
Negative (*n* = 34)	2	32
	rATP-β plus rGroEL	
	Positive	Negative
Positive (*n* = 34)	30	4
Negative (*n* = 34)	3	31
	rLemA	
	Positive	Negative
Positive (*n* = 36)	33	3
Negative (*n* = 34)	15	19
	rSucB	
	Positive	Negative
Positive (*n* = 36)	26	10
Negative (*n* = 34)	11	23
	rVirB5	
	Positive	Negative
Positive (*n* = 36)	26	10
Negative (*n* = 34)	10	24

^*a*^ Group I dogs (n = 36) were naturally infected with *Bartonella *spp. All Group I dogs were *B. henselae* IFA seroreactive (IFA titer ≥1:64). Group II dogs consisted of 34 *Bartonella* spp. IFA-negative and PCR-negative control dogs. For the rATP-β plus rGroEL ELISA, 34 Group I dogs were used due to inadequate serum volumes from two dogs.

## DISCUSSION

In the present study, we describe the development and validation of a reliable ELISA assay for the diagnosis of canine bartonelloses, employing selected regions of *B. henselae* immunodominant proteins ATP-β and GroEL. Based upon the seroreactivity results obtained in this study, rATP-β and rGroEL represented the most sensitive and specific candidate peptide targets for utilization in a canine diagnostic ELISA assay. In this study, rGroEL resulted in 83% sensitivity and 94% specificity at a cutoff OD value of 0.439 and an AUC score of 0.93 (95% CI 0.87–0.99), while the sensitivity and specificity of recombinant rATP-β were 69% and 94% at a cutoff value of 0.565. These findings suggest that these proteins are suitable targets for documenting *Bartonella* exposure in dogs. The combination of rATP-β with rGroEL could potentially further improve both the sensitivity and specificity of an ELISA assay. Based on B-cell epitopes, beta-turn, surface accessibility, antigenicity, and hydrophilicity prediction (http://tools.iedb.org/bcell/), *B. henselae* F0F1 ATP synthase subunit beta (NCBI Reference Sequence WP_011181298.1; ranging from 109 to 265 amino acids) and *B. henselae* GroEL (NCBI Reference Sequence: WP_011181151.1; ranging from 193 to 370 amino acids) appeared to be potentially reliable targets for the diagnosis of *Bartonella* infection in dogs. These immunodominant peptide regions of GroEL and ATP-β, represented by the red box in Fig. 2, generated diagnostically acceptable sensitivity and specificity performances.

To our knowledge, *B. henselae* immunodominant proteins have not been extensively studied for the diagnosis of *Bartonella* infection in dogs. We reported for the first time in this study the diagnostic utility of five *Bartonella* immunodominant proteins: ATP-β, GroEL, LemA, SucB, and VirB5 for the diagnosis of canine bartonelloses. Studies involving cats and humans have reported potential diagnostic utility for several *Bartonella* immunodominant proteins assessed in our study, such as ATP-β, GroEL, LemA, SucB, and VirB5 (19–21, 24, 26, 32, 33). A previous study utilizing a microarray comprised of proteins that were expressed from 96% of predicted ORFs encoded by the *B. henselae* genome to profile antibody response in naturally infected and uninfected cats also found similar immunodominant proteins, including ATP-β, GroEL, LemA, and SucB (24). Consistent with these previous publications, using MALDI-TOF-MS analysis of WB reactive bands from our WB study (8), ATP-β and GroEL were again identified as immunodominant proteins of *B. henselae*. A previous study investigating the proteome of *B. henselae* using 2D SDS-PAGE and MALDI-TOF-MS identified 11 immunodominant proteins, including SucB, as recognized by human sera from patients with clinically suspected and serologically confirmed *B. henselae* infection (32). In a previous study (32), GroEL was strongly reactive with probed human patient sera; however, it was not thought to represent a specific *B. henselae* seromarker due to weak seroreactivity with control sera. Another study identified GroES, BepA, and GroEL as strongly reactive antigens in human *B. henselae* IFA-positive sera (26). Immunoblot analysis results of a recombinant VirB5 17 kDa protein using human sera from CSD cases found a correlation with the *Bartonella* spp. IFA results, suggesting potential diagnostic utility of this protein for the development of more specific diagnostic assays for bartonelloses (20). In the context of canine bartonelloses, 26 of 36 Group I infected dogs and 10 of 34 Group II control dogs were seroreactive to rVirB5, indicating a lack of sensitivity and specificity of this target when testing dog sera. In summary, published studies involving the use of *Bartonella* immunodominant proteins for diagnostic purposes have reported variable sensitivity and specificity. Several factors, such as differences in antigen selection and preparation, variability in the expression of *Bartonella* membrane proteins after vector, bite, or scratch transmission, variability when testing patients during the acute stage compared to patients that are chronically infected, and *Bartonella*’s ability to downregulate gene expression and thereby evade the host humoral response, are all factors that likely contribute to the variability in diagnostic sensitivity and specificity results from different laboratories.

**Fig 7:**
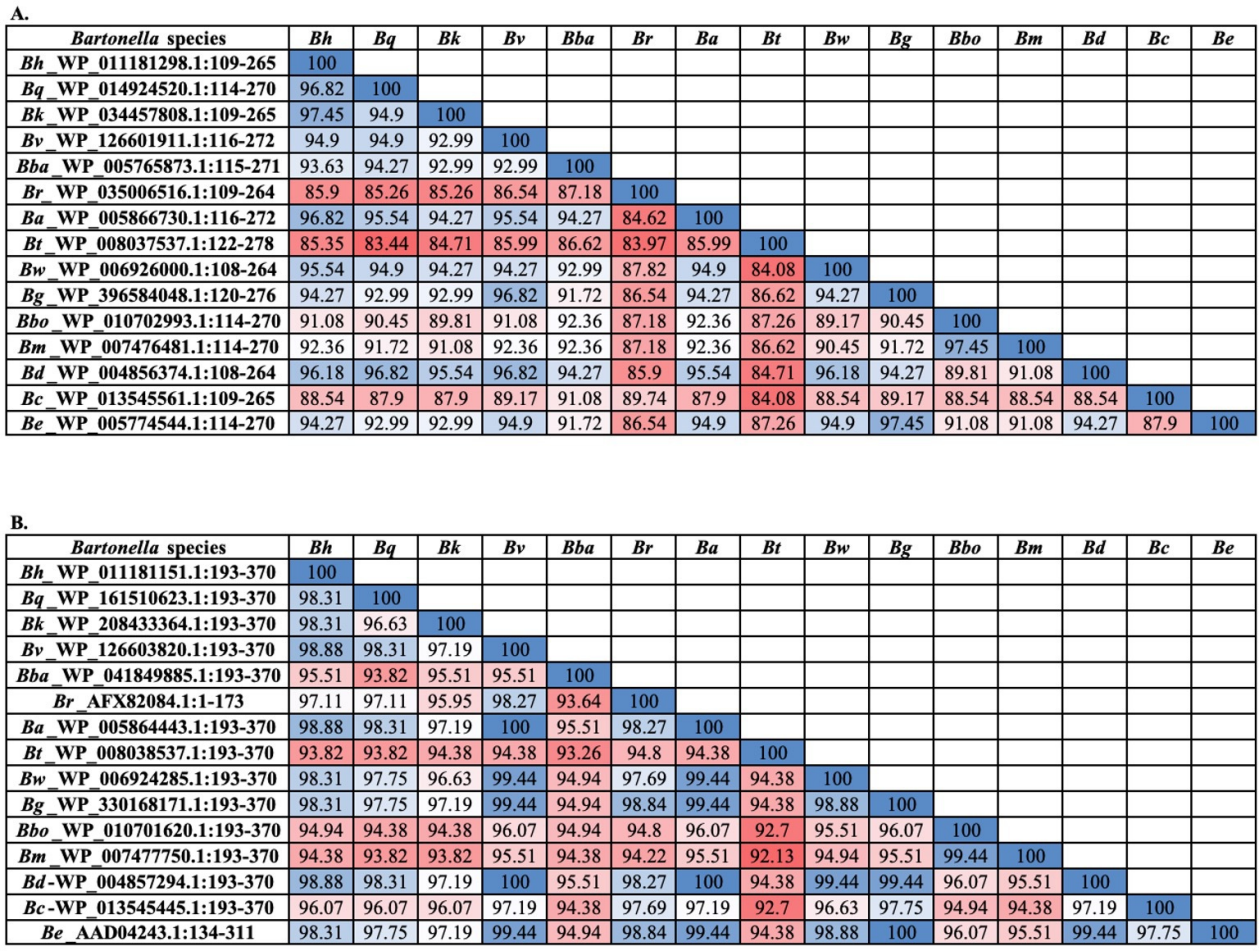
Amino acid sequence-based homology percent identity matrix analysis for the selected region of *B. henselae* ATP-β (**A**) and GroEL (**B**) with medically important dog and human *Bartonella* species. The amino acid region of *B. henelae* ATP-β and GroEL that was selected for ELISA testing in this study was aligned with reference *Bartonella* sequences. Percent identity matrix of proteins was obtained from multiple sequence alignment using Clustal Omega. Values in the box represent the sequence homology in percentage. Colors correspond to the percentage identity with high values (blue), middle values (white), and low values (red). *Bh*, *Bartonella henselae; Bq*, *B. quintanta; Bk*, *B. koehlerae; Bv*, *B. vinsonii*, *Bba*, *B. bacilliformis; Br*, *B. rochalimae; Ba*, *B. alsatica; Bt*, *B. tamiae; Bw*, *Candidatus Bartonella washoeensis; Bg*, *B. grahamii; Bbo*, *B. bovis; Bm*, *B. melophagi; Bd, B. doshiae; Bc*, *B. clarridgeiae; Be*, *B. elizabethae*.

BLAST searches indicate *B. henselae* full-length ATP-β shares 86% to 96.99% identity with other *Bartonella* spp. (86% with *Bartonella* sp. HY038; NCBI Reference Sequence: WP_182417869.1 and more than 87% with clinically important *Bartonella* spp., including *B. bacilliformis* (87.52%)*, B. vinsonii* (91.08%)*, B. quintana* (92.25%), and *B. koehlerae* (96.05%). *Bartonella henselae* full-length GroEL shares at least 91% identity with other *Bartonella* spp. and a high degree of identity (up to 91%) with chaperonin GroEL proteins from other bacteria, including *Brucella* (up to 89% identity; NCBI Reference Sequence: WP_188820551.1). These results indicate that the diagnostic utility of the entire length of ATP-β and GroEL may suffer from significant cross-reactivity and questionable sensitivity for diagnostic purposes. Low sensitivity and serological cross-reactivity between *Bartonella* species and other bacterial pathogens would clearly complicate the accurate diagnosis of Bartonella infection. Therefore, we used an immunoproteomic approach to select antigenic regions of ATP-β and GroEL for the diagnosis of bartonelloses in dogs. The selected region of ATP-β in this study shares identity from 85% (*B. tamiae*: NCBI Reference Number WP_008037537.1:122-278) to 100% with *Bartonella* spp, including medically important dog and human *Bartonella* spp., such as *B. bacilliformis* (94%; NCBI Reference Sequence: WP_005765873.1), *B. vinsonii* (95%; NCBI Reference Sequence: WP_126601911.1), *B. quintana* (97%; NCBI Reference Sequence: WP_014924520.1), 97% *B. koehlerae* (97%; NCBI Reference Sequence: WP_034457808.1), and 100% with *B. henselae* (NCBI Reference Sequence: WP_011181298.1; Fig. 7A). The selected region of GroEL in this study shares 94%–100% identity with GroEL of clinically important canine and human *Bartonella* species (Fig. 7B). These findings suggest that the selected region of ATP-β and GroEL used in this study might be appropriate for the detection of antibodies induced by infection with other *Bartonella* spp. in dogs. Further optimization of ATP-β and GroEL by epitope identification, mapping, and profiling using sera from *Bartonella*-infected dogs is needed to further enhance the diagnostic sensitivity and specificity of ATP-β- and GroEL-based ELISA for bartonelloses.

To assess ELISA specificity and cross-reactivity, we tested 34 Group II control dogs, 10 of which were IFA seropositive (IFA titer of ≥1:64) to *R. rickettsii*, an alpha proteobacterium phylogenetically closely related to *Bartonella* spp. Previous studies involving dogs experimentally infected with *R. rickettsii* documented no cross-reactivity between *R. rickettsii* and *Bartonella* antigens (34, 35). In concordance with the findings of previous studies (11, 34, 35), lack of ELISA seropositivity in seven of 10 *R. rickettsii* IFA seropositive Group II control dogs on rATP-β, rGroEL, and rATP-β plus rGroEL ELISA supports the use of rATP-β and rGroEL in serodiagnostic tests for canine bartonelloses that are unlikely to yield false positives due to *R. rickettsii*.

Limitations of this study include the lack of historical and clinical information for diagnostic serum specimens from sick dogs that were used to assess the sensitivity and specificity of each of the ELISA assays. The number of sera from infected dogs represented a relatively small sample size. Considering the somewhat ubiquitous nature of fleas, cats, and other sources of *Bartonella* spp. infections, dogs in the naïve control groups could have been exposed to *Bartonella* spp. despite negative *Bartonella* IFA and PCR testing. Despite being the current “gold standard” reference diagnostic assay used in this study, *B. henselae* IFA has sensitivity and, to a lesser extent, specificity limitations. Sera obtained from specific pathogen-free dogs maintained in a vector-free facility would serve as a more optimal source of negative control sera; however, based upon a prior publication from our laboratory, the possibility of *Bartonella* exposure among dogs obtained from research vendors is problematic for experimental and research studies due to their prior exposure to *Bartonella* spp. (36). Another limitation of this study is the lack of validation for the serological cross-reactivity of these recombinant proteins with sera from dogs exposed to other pathogens. Further studies are warranted to assess potential cross-reactivity of these recombinant proteins with sera from dogs infected with other canine pathogens, including phylogenetically related pathogens to *Bartonella* spp., such as *Brucella canis*. Also, additional research testing of sera from dogs that are *Bartonella* PCR positive or PCR positive after *Bartonella* alpha proteobacteria enrichment blood culture is needed to assess assay sensitivity. The study is limited by the use of IFA assay as the reference standard for calculating the sensitivity and specificity, despite its diagnostic limitations. A limitation of this study is that ELISA testing of serum samples from Group I and Group II dogs was performed without complete blinding, which could potentially introduce bias. However, this is unlikely to impact the accuracy or reliability of the ELISA assay results, as appropriate precautions were taken during both the ELISA testing and analysis phases. Another limitation of this study is the potential impact of expressing *B. henselae* proteins in the *E. coli* expression system, which may affect proper protein folding and antigenicity. Further structural or functional validation of antigen conformation may be needed to optimize assay specificity and sensitivity.

In conclusion, ATP-β and GroEL may serve as cost-effective ELISA antigens for the serodiagnosis of *Bartonella* infection in dogs. Availability of a rapid in-house diagnostic test would facilitate a more rapid diagnosis and the initiation of prompt treatment. Assays with improved sensitivity and specificity would also improve the conclusions derived from dog seroepidemiological surveys. We developed novel rATP-β, rGroEL, or a combination of rATP-β plus rGroEL-based ELISA assays that may provide high-throughput, rapid, and cost-effective serodiagnostic results compared to the tedious reference IFA assay for diagnosis of canine bartonelloses. Further epitope mapping and screening of *B. henselae* ATP-β and GroEL proteins by immunoproteomic approaches using sera from large numbers of *Bartonella-*infected dogs and well-defined control cohorts is needed to further characterize the sensitivity, specificity, or cross-reactivity of these assays.

## Data Availability

The data that support the findings of this study are openly available in Dryad at https://doi.org/10.5061/dryad.9zw3r22t9.

## SUPPLEMENTAL MATERIAL

Supplemental tables jcm.00267-25-s0001.pdf Tables S1 to S3.

ASM does not own the copyrights to Supplemental Material that may be linked to, or accessed through, an article. The authors have granted ASM a non-exclusive, world-wide license to publish the Supplemental Material files. Please contact the corresponding author directly for reuse.
