# Supplementary material for: Development and validation of enzyme-linked immunosorbent assays for the serodiagnosis of canine bartonelloses
Source: J Clin Microbiol. 2025 Nov 26;63(12):e00267-25. doi: 10.1128/jcm.00267-25 (PMC12710310; doi:10.1128/jcm.00267-25)
Supplement: Supplemental tables — Tables S1 to S3. [file jcm.00267-25-s0001.pdf]

Supplementary materials

Supplementary Table S1. *Bartonella* and canine vector-borne disease (CVBD) testing results for dogs' samples used for comparative ELISA testing in this study. Canine comprehensive panel testing including *Bartonella* tests were performed at Vector Borne Disease Diagnostic Laboratory (VBDDL) and Intracellular Pathogen Research Laboratory (IPRL) at the North Carolina State University, College of Veterinary medicine (NCSU-CVM). BAPGM test results are not available for all Group I and II dogs due to inadequate blood volume.
Bart.= *Bartonella* spp., POS= positive; NEG= negative; NT= not tested; n/a = not available; *Bh* = B. *henselae* ; *Bvb* T1= *B. vinsonii* subsp. *berkhoffii* (*Bvb*) genotype I; Rr = *Rickettsia rickettsii*; IFA= Immunofluorescent antibody assay; CVBD PCR = PCR testing for *Babesia, Ehrlichia, Anaplasma, Rickettsia*, hemotropic *Mycoplasma*, and *Leishmania* spp.; CVBD serology = IFA testing for *Rickettsia rickettsii*, *Ehrlichia canis*, *Babesia canis*, and *Babesia gibsoni* plus ELISA testing (SNAP 4Dx PLUS ELISA, IDEXX Laboratories, Westbrook, Maine) for *Anaplasma phagocytophilum*, *Anaplasma platys*, *Borrelia burgdorferi, Ehrlichia canis*, and *Ehrlichia ewingii*. Detailed methods for *Bartonella* PCR, BAPGM (*Bartonella* Alpha Proteobacteria Growth Medium) enrichment blood culture, and IFA serological panel used to test these study samples have been published previously [36].

Of the four dogs positive for CVBD by serology, one dog was seropositive to *Babesia canis* (IFA titer 1:1024), *Babesia gibsoni* (IFA titer 1:4096), and *Rickettsia rickettsii* (IFA titer 1:128), one dog was seropositive to *Ehrlichia canis* (IFA titer 1:2048), one dog was seropositive to *Babesia canis* (IFA titer 1:2048) and *Babesia gibsoni* (IFA titer 1:2048), and the remaining dog was seropositive to *Babesia canis* (IFA titer 1:64 and *Rickettsia rickettsii* (IFA titer 1:512). *represents two (2*) of these four dogs. A cutoff titer of ≥1:64 was used to define an IFA seropositive titer.

Molecular tests	Serological tests	Test results	Group I (*Bartonella* spp. naturally infected dogs (Bart. IFA POS), n=36)	Group II (*Bartonella* spp. PCR NEG and IFA NEG dogs (controls, n=34)
***Bartonella* spp. qPCR (Strain)**		POS	3 [2 (*Bv);* 1 (*Bh*)]	0
		NEG	33	34
**BAPGM Enrichment Culture (n)**		POS	0	0
		NEG	8	21
		NT	28	13
**CVBD PCR**		POS	0	0
		NEG	24	16
		NT	12	18
	***Bh* IFA**	POS	36	0
		NEG	0	34
	***Bvb* T1 IFA**	POS	23	0
		NEG	13	34
	***Bk* IFA**	POS	32	0
		NEG	3	34
	***Rr* IFA**	POS	2*	10
		NEG	26	6
		NT	8	18
	**CVBD serology**	POS	4	0
		NEG	24	16
		NT	8	18

Supplementary Table S2. Sensitivity and specificity of *Bartonella henselae* recombinant proteins-based ELISA for the diagnosis of *Bartonella* infection at different cutoff values. Sensitivity and specificity were calculated using sera from Group I dogs (*Bartonella* spp. naturally infected dogs; n=36) and Group II dogs (34 *Bartonella spp.* IFA negative and PCR negative control dogs). All Group I dogs were *B. henselae* IFA seroreactive (IFA titer ≥1:64). For the rATP-β plus rGroEL ELISA, 34 Group I dogs were used due to inadequate serum volumes from two dogs. Optimal density (OD) cutoff values were determined to maximize Youden index as previously described. *represents the ELISA cutoff OD values at maximum Youden index.

Proteins	Cutoff OD	Sensitivity (95% CI)	Specificity (95% CI)
**rATP-β**	0.417	69% (51.89% to 83.65%)	85% (68.94% to 95.05%)
	0.565*	69% (51.89% to 83.65%)	94% (80.32% to 99.28%)
	0.643	61% (43.46% to 76.86%)	97% (84.67% to 99.93%)
	0.765	50% (32.92% to 67.08%	100% (89.72% to 100.00%)
**rGroEL**	0.346	89% (73.94% to 96.89%)	82% (65.47% to 93.24%)
	0.439*	83% (67.19% to 93.63%)	94% (80.32% to 99.28%)
	0.661	61% (43.46% to 76.86%)	97% (84.67% to 99.93%)
**rATP-β plus GroEL**	0.406	88% (72.55% to 96.70%)	79% (62.10% to 91.30%)
	0.505*	88% (72.55% to 96.70%)	91% (76.32% to 98.14%)
	1.195	74% (55.64% to 87.12%)	100% (89.72% to 100.00%)
**rLemA**	0.136*	92% (77.53% to 98.25%)	56% (37.89% to 72.81%)
	0.216	69% (51.89% to 83.65%)	59% (40.70% to 75.35%)
	0.669	33% (18.56% to 50.97%)	71% (52.52% to 84.90%)
**rSucB**	0.501	92% (77.53% to 98.25%)	38% (22.17% to 56.44%)
	0.785*	72% (54.81% to 85.80%)	68% (49.47% to 82.61%)
	1.082	39% (23.14% to 56.54%)	88% (72.55% to 96.70%)
**rVirB5**	0.403	86% (70.50% to 95.33%)	50% (32.43% to 67.57%)
	0.525*	72% (54.81% to 85.80%)	71% (52.52% to 84.90%)
	0.705	53% (35.49% to 69.59%)	79% (62.10% to 91.30%)
	0.977	31% (16.35% to 48.11%)	97% (84.67% to 99.93%)

Supplementary Table S3. ELISA optical density (OD) readings for Group I dogs (*Bartonella* spp. naturally infected dogs; n=36) and Group II dogs (34 *Bartonella spp.* IFA negative and PCR negative control dogs). All samples were tested in duplicate. The average absorbance value was calculated for each set of duplicate samples.

Dog Groups	Dog ID	ELISA Optical Density (OD) readings at 450nm					
		rATP-β ELISA	rGroEL ELISA	rLemA ELISA	rSucB ELISA	rVirB5 ELISA	rATP-β plus rGroEL ELISA
Group I (naturally infected with *Bartonella spp.*, *n*=36)	1	0.565	0.726	0.36	0.527	0.902	1.4835
	2	0.7655	0.335	0.322	0.84	0.4035	1.5835
	3	0.8765	0.593	0.9645	0.578	0.7255	1.9825
	4	0.6775	0.7625	0.5855	0.933	0.413	0.6615
	5	0.0855	0.6125	0.4895	0.393	0.431	2.1925
	6	0.657	0.5695	0.669	0.633	0.5595	2.196
	7	0.643	0.526	0.2815	0.501	0.508	2.2645
	8	0.98	0.931	0.181	0.804	0.57	1.652
	9	0.936	1.193	1.156	0.868	1.537	N/A
	10	0.183	0.436	0.057	0.9295	0.319	0.5145
	11	0.769	0.8515	0.4115	1.0885	1.1075	2.523
	12	0.153	0.8315	0.1105	1.0655	0.5255	1.195
	13	0.604	0.8895	0.2025	0.7585	0.844	2.14
	14	0.091	0.439	0.197	0.518	0.709	1.389
	15	1.029	1.2175	0.684	1.4435	1.5605	N/A
	16	0.2525	1.4615	0.1415	0.9495	0.317	2.0425
	17	0.199	0.149	0.142	0.6925	0.177	0.055
	18	0.683	1.9445	0.348	1.7695	1.13	2.5055
	19	0.293	1.5945	0.3145	0.8635	0.685	1.774
	20	1.584	0.7505	0.2685	1.696	0.6685	1.3235
	21	0.0445	0.532	0.07	0.356	0.149	0.094
	22	1.1065	0.456	0.228	0.3235	0.609	1.6735
	23	0.4115	1.3215	0.216	1.033	0.771	2.356
	24	0.9025	0.4115	0.721	1.141	0.364	1.0905
	25	0.5985	1.349	0.1975	1.133	1.1025	2.5045
	26	1.156	1.7065	0.161	1.8395	1.828	2.513
	27	1.0545	0.9435	0.267	1.4125	0.661	1.6345
	28	0.2565	0.285	0.136	1.0825	0.454	0.3495
	29	0.163	0.888	0.302	0.785	0.918	2.9695
	30	1.156	0.661	1.379	1.782	0.977	0.6215
	31	1.2135	1.737	1.4365	1.8285	1.2675	1.9935
	32	1.2945	1.5775	1.6375	1.9405	1.0825	2.4885

	33	0.9	0.201	1.119	1.085	0.7705	0.129
	34	0.928	0.615	0.826	1.1195	1.045	0.505
	35	0.9085	0.833	0.8145	0.9335	1.0715	1.3305
	36	0.825	0.693	0.8105	0.89	0.716	1.692
**Group II (*Bartonella* spp. PCR negative and IFA negative control dogs, n=34)**	1	0.337	0.154	0.566	0.548	0.103	0.146
	2	0.281	0.344	0.095	0.699	0.423	0.471
	3	0.287	0.346	0.097	0.7	0.423	0.2315
	4	0.1195	0.3855	0.431	1.185	0.483	0.2
	5	0.147	0.112	0.062	0.7415	0.91	0.128
	6	0.2795	0.222	0.1185	0.8265	0.524	0.232
	7	0.3905	0.4375	0.2045	1.1035	0.504	0.4
	8	0.264	0.332	0.0855	1.0635	0.61	0.3895
	9	0.112	0.0815	0.053	0.218	0.193	0.103
	10	0.101	0.0365	0.041	0.259	0.22	0.077
	11	0.1635	0.1885	0.6925	0.081	0.524	0.326
	12	0.144	0.1595	0.8105	0.1035	0.7055	0.251
	13	0.1695	0.1555	0.8185	0.097	0.354	0.3065
	14	0.3895	0.4145	1.2385	0.127	0.4045	0.4025
	15	0.169	0.114	0.5155	0.081	0.1985	0.236
	16	0.2165	0.2865	0.085	0.6655	0.722	0.4445
	17	0.1305	0.05	0.0085	0.2325	0.2035	0.137
	18	0.158	0.153	0.026	0.675	0.2165	0.398
	19	0.2765	0.3235	0.091	0.5675	0.2535	0.6975
	20	0.056	0.063	0.0135	0.303	0.1065	0.079
	21	0.217	0.2895	0.0915	0.456	0.2065	0.3625
	22	0.041	0.0385	0.025	0.691	0.0665	0.052
	23	0.035	0.015	0.002	0.216	0.084	0.306
	24	0.231	0.139	0.077	0.909	0.118	0.315
	25	0.2615	0.3225	1.278	0.9335	0.705	0.289
	26	0.3085	0.651	1.119	0.8795	0.5685	0.5625
	27	0.4615	0.2865	1.254	1.0275	0.9445	0.382
	28	0.607	0.2525	1.3685	1.3685	1.161	0.406
	29	0.2455	0.0315	0.651	0.651	0.3985	0.477
	30	0.747	0.6925	1.46	1.46	0.853	1.1055
	31	0.414	0.1525	1.328	0.9695	0.8425	0.3845
	32	0.449	0.279	0.044	0.246	0.2115	0.398
	33	0.4175	0.234	0.088	0.7645	0.3495	0.141
	34	0.189	0.279	0.0105	0.1225	0.2115	0.0855
